# Pediatric Langerhans cell histiocytosis of the temporal bone: clinical and imaging studies of 27 cases

**DOI:** 10.1186/s12957-018-1366-x

**Published:** 2018-03-27

**Authors:** Hui Zheng, Zhengrong Xia, Wenjun Cao, Yun Feng, Shuxian Chen, Yu-Hua Li, Deng-Bin Wang

**Affiliations:** Department of Radiology, Xinhua Hospital affiliated to Shanghai Jiao Tong University School of Medicine, Room 203, Building 5, No.1665 Kongjiang Road, Shanghai City, 200092 Yangpu District China

**Keywords:** Langerhans cell histiocytosis, Temporal bone, Children, Imaging

## Abstract

**Background:**

We aimed to evaluate the clinical and imaging presentations of Langerhans cell histiocytosis (LCH) in the pediatric temporal bone.

**Methods:**

This retrospective study included 27 pediatric cases with pathological confirmed LCH of the temporal bone. The clinical and imaging features of the cases were analyzed. The involvement of ossicular chain and otic capsule was also evaluated.

**Results:**

A total of 38 lesions (27 cases) with 11 bilateral involvement were identified. For the 27 cases, the most common complaint was periauricular swelling (12/27, 44.4%), followed by otorrhea (9/27, 33.3%) and otalgia (5/27, 18.2%). The mastoid process was the most common involved subsite (31/38, 81.6%) among the 38 lesions. Ten (26.3%, 10/38) lesions belonged to the group of the diffuse involvement, 22 (57.9%, 22/38) were divided into the group of partial involvement and six (15.8%,6/38) localized lesions with punched-out appearance. Erosion of ossicular chains and otic capsule were found in three and seven lesions respectively.

**Conclusion:**

The results indicate that the most common subsite for LCH of the pediatric temporal bone was the mastoid process. The location and extent of pediatric LCH of the temporal bone varied a lot between each other. The ossicular chains usually remain intact and the erosion of otic capsule can occur in some lesions.

## Background

Langerhans cell histocytosis (LCH) is a disease characterized by accumulation of Langerhans cells. The immunohistochemical feature of LCH is positive CD1a and/or S100 antigen [1, 2]. It has a tendency to affect young adults and children. The annual morbidity of this disease is about three to five per million per year in the group of children [3, 4]. Any organ or system can be affected; flat bones especially the skull are the most frequently involved. The other possibly involved organs are the skin, pituitary, liver, spleen, lung and lymph nodes [4]. Disease involvement of risk organs such as the liver and spleen is a sign of poor prognosis [5, 6]. Based on the number of lesions and the involved systems, LCH can be divided into three stages: single system (stage 1), low-risk multisystem (stage 2), and multisystem with risk-organ involvement (stage 3) [7]. In cases with single-system LCH, it may involve a single site or multiple sites of one organ/system. Stage 2 lesion is defined as involvement of two or more organs/systems without involvement of risk-organs (bone marrow, liver and/or spleen). It will be recognized as stage 3 if any risk organ is involved.

About 50~ 80% of pediatric LCH is found in the head and neck regions. The temporal bone is involved approximately 15 to 60% of cases in this region [8]. Otologic presentations include mastoid swelling or temporal bone mass, otalgia, and otorrhea. The otologic findings are very similar to the otitis media, otitis externa, cholesteatoma, and the other conditions; for this reason, the diagnosis is usually delayed [9]. There are a series of treatment protocols that can be chosen once the diagnosis is made. It includes surgery only, chemotherapy after diagnostic biopsy, systemic steroids, or a combination therapy depending on the extent of the disease [6].

Therefore, understanding of the extent of the lesion and possible pathological pattern is helpful for treatment planning. The imaging modalities primarily CT and MR can manifest these important information accurately. In order to find some specific clinical and imaging characteristics, we retrospectively reviewed the clinical symptom, physical and auriscopic imaging and pathological findings of pediatric LCH of the temporal bone.

## Methods

### Patient selection

Following institutional approval, we used a radiology data bank by the key words temporal bone lesion to find cases with possible LCH affecting the temporal bone. And then checked the histopathologic results to recruit the cases with LCH. Each imaging study of LCH was reviewed to ensure that CT and/or MR images were available. Data collection included demographics, clinical presentation, imaging, and histopathologic findings of the cases. Only the cases with thin slice CT and MR studies of internal auditory canal were enrolled in our study. The images with evident artifact were excluded. For this retrospective study only used the previous database of the patients, the informed consent was waived.

### Imaging protocol

Temporal bone high-resolution CT (HRCT) was obtained on a 64-section CT scanner (Siemens Somatom Definition) using the protocols as following: 120 KVp, 230 mAs, thickness 0.67 mm, spacing 0 mm. Coronal reformation was included for evaluating the small bony structures such as the semicircular canals.

Each MR scan was obtained using a 3 T MR unit with an eight-channel phase-array head coil (Signa HDxt; GE Healthcare). The MRI sequences consisted of axial T1- and T2-weighted images (T1WI and T2WI) with thickness of 2 mm. The axial and coronal contrast-enhanced (CE)-T1WIs were obtained after intravenous administration of gadolinium-diethylenetriamine pentaacetic acid (Gd-DTPA, Magnevist, Bayer-Schering Pharma AG).

#### Anatomical subsite classification

**Fig. 1:**
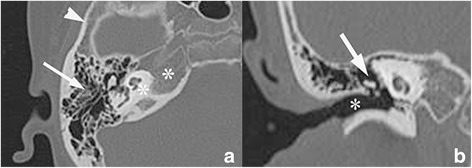
Axial HRCT of the temporal bone shows the five regions used to categorize the lesion (mastoid process: long arrow in **a**; petrous apex: star in **a**; squamas: arrowhead in **a**; external auditory canal: star in **b**; middle ear: long arrow in **b**

A subsite classification was made to describe the accurate location and extent of the lesion within the temporal bone. We divided the temporal bone into five subsites including the mastoid process, external auditory canal, middle ear, squamas, and petrous apex (including otic capsule) (Fig. 1). The degree of involvement was classified by a scale. If the specific part of the temporal bone was a little bit affected by the lesion, it would get the score of 0.5. And when the specific part was partial (less than a half) or diffuse (more than a half) involvement, the score was 1 or 2 for the two situations.

According to the range of bony destruction, we divided the lesions into three groups. Group 1 was diffuse pattern with complete destruction of at least three subsites with total 2 points. Partial destructive pattern was defined as partial involvement of the temporal bone and mastoid process must be included. Localized punched-out pattern had focal erosion of the temporal bone. And we also evaluated erosion of otic capsule and ossicular chain as sparing or affected.

The images were independently reviewed by two experienced neuroradiologist (W.J Cao and Y.H Li with 6 and 15 years of clinical experience in temporal bone imaging respectively).

### Statistical analysis

Continuous data were summarized as median and ranges, and the categorical data were calculated using frequency counts and percentages. Subsite scores were compared with a *kappa* value to evaluate the interobserver agreement. *P* value < 0.05 was statistically significant.

## Results

### Demographics and clinical presentation

A total of 27 cases with 11 bilateral lesions were identified by reviewing medical records from January 2010 to March 2016. The median age at presentation was 24 months (range 6 months to 4 years). 44.4% (12/27) were diagnosed before the age of 2 years old. Male to female ratio was 13:14. 51.9% (14/27) belonged to stage 1, 33.3% (9/27) stage 2, and 14.8% (4/27) stage 3.

Periauricular swelling was the most common complaint (44.4%, 12/27), followed by otorrhea (33.3%, 9/27) and otalgia (22.2%, 6/27). 74.1% (20/27) were found to have temporal bone lesions at initial presentation. The most common physical examination finding was hard and immobile periauricular mass (37%, 10/27). The auriscopic examination showed neoformation or granulation tissue in the external auditory canal (EAC) (22.2%, 6/27) and EAC stenosis (14.8%, 4/27).

25.9% (7/27) of cases manifested abnormality of the other system at onset. It includes jaundice, diabetes insipidus, and cutaneous erythema. Six cases presented low-grade fever as a systemic symptom. 11.1% (3/27) had intracranial involvement; all of the three cases presented as diabetes insipidus. The clinical presentation of the cases is summarized in Table 1.

**Table 1:** The clinical presentation of disease

Clinical features	No. (%)
Symptom, *n* = 27	
Periauricular swelling	12 (44.4)
Otorrhea	9 (33.3)
Otalgia	6(22.2)
Physical and auriscopic examination	
Hard and immobile mass	10 (37.0)
External or media otitis	7 (25.9)
Granulation tissue	6 (22.2)
EAC stenosis	4 (14.8)
The other systemic symptom	
Cutaneous erythema	3 (11.1)
Diabetes Insipidus	3 (11.1)
Mass in the other region	2 (7.4)
Lower back pain	1 (3.7)
Cheek swelling	1 (3.7)

*EAC* external auditory canal

### Imaging manifestations

CT and MR images were available for 17 children. Seven cases only had CT studies, and three children only had MR studies.

For we have altogether 38 lesions in this series, the highest score for a subsite of the temporal bone was 76 (a total of 2 points each). The interobserver agreement by a weighed *κ* was very good for all of the subsites (*P<*0.05 for all of the comparisons). Mastoid was the most commonly involved region (81.6%, 31/38) with highest score of 41.5 followed by squama (78.9%, 30/38) with a score of 33.5. External and middle ear got the same score and ratio (scores were 27 points for each other). The detailed scoring and ratio of involvement is presented in Table 2. Ten (26.3%, 10/38) lesions belonged to the group of diffuse pattern, 22 (57.9%, 22/38) partial involvement, and six (15.8%, 6/38) focal pattern. There are no differences in subsite score of the temporal bone among the three-stage groups. The most common involved subsite for the three groups was all the mastoid process. The scoring of all three groups was present in Table 3. The group of stage 1 was more likely to involve the petrous apex than the other two groups, and the group of stage 2 was less likely to involve the external ear.

**Table 2:** Subsite scores for LCH of the temporal bone

Subsite	Points	Ratio of involvement (%)
Mastoid	41.5	53. 2
Squama	33.5	42.9
External ear	27	35.5
Middle ear	27	34.9
Petrous apex	10.5	26.3

**Table 3:** Subsite scores and the ratio of involvement for the three-stage groups

Group	Mastoid	Squama	External ear	Middle ear	Petrous apex
Stage 1	18	16.5	17.5	17	8.5
	23.2%	21.3%	22.6%	21.9%	11.0%
Stage 2	13	13	6	8.5	1
	31.3%	31.3%	14.5%	20.5%	2.4%
Stage 3	8	3	5	5	1
	36.4%	13.6%	22.7%	22.7%	4.6%

**Fig. 2:**
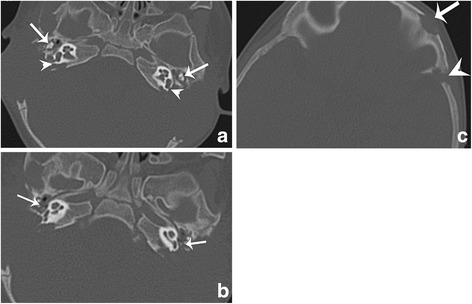
Axial HRCT of the temporal bone demonstrates diffuse and symmetric destruction of the bilateral temporal bone. The ossicular chains remain intact (arrow in **a**). Both of the posterior SCCs are completely destructed (arrowhead). The smallest bone stapes are still in good condition even if they are surrounded by LCH tissue (arrow in **b**). Axial CT scan also shows the erosion of the frontal bone and squama (**c**)

In lesions with diffuse pattern, CT showed diffuse and complete destruction of the temporal bone upon the removal of it, the margin was sharp and irregular. And in three cases, the bony destruction was bilateral and symmetric (Fig. 2). On MR imaging, it showed isointensity on T1WI, heterogeneous on T2WI and heterogeneous enhancement after administration of contrast. Part of the mass showed very low signal intensity on T2WI, and it was identified as intratumoral hemorrhage on pathological examination. Four of the 10 lesions demonstrated radiographic signs of labyrinth involvement. The specific involvement of otic capsule is summarized in Table 4. There are two lesions whose ossicular chain completely disappeared. And in the other eight lesions, the ossicular chain remained intact even when they were completely surrounded by the pathological tissue.

**Table 4:** Involvement of otic capsule

Specific part	No.
Posterior SCC	6
Superior SCC	4
Lateral SCC	3
Vestibule	1
Cochlea	1

*SCC* semicircular canal

**Fig. 3:**
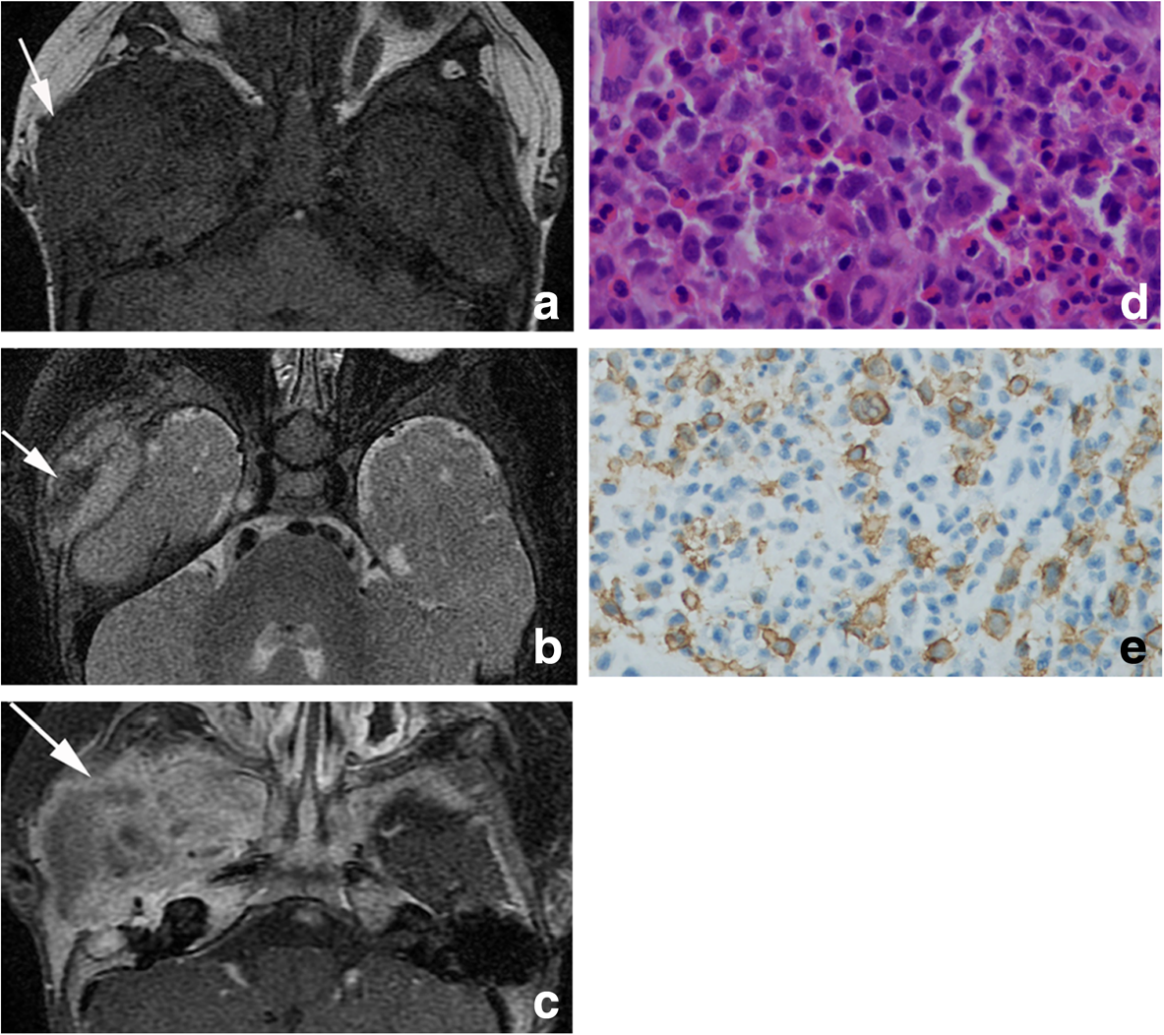
Axial T1-weighted image (**a**) demonstrated a big LCH lesion with isointensity. It shows heterogeneous on T2 fat-saturation MR image for intratumoral hemorrhage (arrow in **b**).On enhanced MR image, it shows heterogenous and avid enhancement (**c**). Hematoxylin–eosin staining (× 400) (**d**) of LCH demonstrates Langerhans cell mixed with eosinophils and multi-nucleated giant cells. Immunohistochemical (× 400) (**e**) demonstrates positive staining for CD1a of the same LCH sample

Among 22 lesions of partial pattern, 10 were centered in the mastoid process. On CT images, it manifested as complete destructive of the mastoid process or squama with irregular margin and sharp boundary. On MR images, it showed as hypointensity or isointensity on T1W and heterogeneous on T2W. Four lesions demonstrated low signal intensity on T2WI for intratumoral hemorrhage (Fig. 3). Among the lesions, three had involvement of otic capsule (Table 4). Only one lesion had slightly bony absorption of the incus body.

**Fig. 4:**
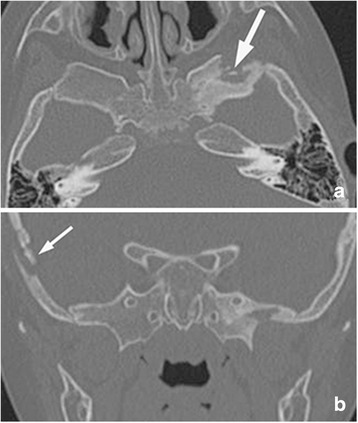
Axial CT shows focal bony erosion of the left greater wing of sphenoid bone with well-defined margin (**a**), which was pathologically confirmed LCH. Focal well-defined lytic lesion with punched-out appearance of squamous part of the temporal bone is clearly demonstrated on CT (**b**)

The localized punched-out pattern is a classic manifestation of skull lesions. We only had six lesions of this type. All of them were centered in the squamous part. It was easily missed on MR images. The ideal modality was high-resolution CT scans with 1-mm thickness. It showed scattered well-defined lytic lesions with punched-out appearance. This pattern was caused by asymmetric destruction of inner and outer cortices (Fig. 4). On MR images, it showed isointensity both on T1- and T2-weighted images, with marked enhancement after administration of contrast.

## Discussion

LCH results from a clonal proliferation of Langerhans cells with dendritic cell features. Whether LCH is a neoplastic or inflammatory disease is a major question to be answered. Now, the finding that driver somatic mutations in BRAF in 55% of cases with LCH and activation of the RAS-RAF-MEK-ERK pathway in nearly all of the cases satisfied evidence for LCH to be a neoplastic disease [10].

LCH can affect any age group and has a predilection to children and young adults. In our series of pediatric LCH in the temporal bone, the media age at presentation was 24 months (range 6 months to 4 years). A male to female ratio 13:14 was different from the literature which stated a male predominance [2, 9]. In the literature, LCH can affect both ears in 25~ 30% of cases [11, 12]. In our series, 11 of 27 cases (40.7%) have bilateral involvement.

### Clinical presentation

For LCH of the temporal bone, there is no characteristic clinical presentation and it varies a lot depending on the site of involvement. The most common involvement site is the mastoid process, in which it is easily extending to the middle and external ears. So a little different from the literature [2, 9], the most common presentation of our cases is periauricular mass (44.4%) followed by otorrhea (33.3%). However, otalgia, polyp in EAC, and EAC stenosis were comparatively less common, which is consistent with literature.

The mass in other parts of the scalp, cutaneous erythema, diabetes insipidus, jaundice, and so on can present as part of multisystemic manifestation [13, 14]. In our series, cutaneous erythema and diabetes insipidus were common findings.

### Subsite classification of the temporal bone

Similar to the findings reported, LCH usually involves several subsites of the temporal bone by either extending directly or multiple involvement [12, 15]. In the study of Fernandez-Latorre et al., there were 12 lesions among total 14 lesions involving mastoid, 9 middle ear, and 2 petrous bone. In this study, we used a subsite classification system to evaluate the extent of the lesion. The score showed that in our 38 lesions the most common involved portion was the mastoid followed by the squama. Consistent with the literature, temporal bone LCH is usually associated with multiple lesions [1, 6, 14], even if unifocal lesions may extensively involve sphenoid, zygomatic bone, or orbit [3, 16, 17].

### Involvement of ossicular chain and otic capsule

We only found several literatures describe the ossicular chain involvement in the case of LCH of the temporal bone [12]. Only several reports mentioned otic capsule erosion and regarded this condition as a rare performance [18]. According to this series, we found that ossicular chain involvement was unusual, but the ratio of otic capsule involvement was not lower. This was a kind of specific appearance and could help in making differentiating from the other conditions.

In our series, even if the ossicular chain was completely surrounded by the lesion, the small bones would be kept intact. In our 38 cases, only two cases’ ossicular chain had completely disappeared and one case with focal bony destruction of incus body. Maybe ossicular bones are resistant to the granulation tissue of LCH because of its density. And the complete or focal destruction of the ossicular chain may associate with chronic otitis media. And in a retrospective review by Saliba et al., only one had signs of inner ear in their 10 cases [2]. Different from the literature, in our study of 38 cases, 7 cases (18.4%) demonstrated with otic capsule erosion. The posterior semicircular canal was the most commonly involved.

### Imaging

On conventional X-ray, LCH lesions involving skull bone showed “punched out” appearance because of unsymmetrical destruction of the inner and outer plates [11]. The erosion of the inner plate is usually more than the outer plate; a beveled edge may be observed. Because the current study recruited cases in recent years, we did not have any cases with plain film. The similar features on plain film can be seen on CT scans. Honestly speaking, in our series, cases with this characteristic manifestation were not common. Only six cases in the localized punched-out pattern group would show double contour sign on HRCT. The squamous part was the most common site. On MR images, it was easily missed if there was no CT to be referred. We had one case who accepted MR examination at the first time after onset of disease. And the primary doctor did not find any abnormity in the MR images, and the superior doctor checked it out. It showed multiple focal abnormity of the bilateral squamous part of the temporal bone and sphenoid bone. The lesions manifested as isointense both on T1- and T2-weighted images and homogeneously marked enhancement after administration of contrast material. The lesions were confirmed by the subsequent HRCT.

The cases of partial and diffuse pattern showed bony destruction of the temporal bone and soft tissue mass both on CT and MR images. It may be manifested as low signal intensity on T2-weighted image in the lesion because of diffuse intratumoral hemorrhage. It could easily manifested accompanied otitis mastoid on MR images as obviously hyperintense on T2-weighted images.

The differential diagnosis for cases with LCH in the pediatric temporal bone is broad depending on the center of the lesion. It may mimic acute and chronic otitis media or cholesteatoma (CH) if the lesion is centered in the middle ear and mastoid process. Lesions involving the petrous apex should consider another malignant entity rhabdomyosarcoma (RMS).

LCH of the mastoid tend to be paracentric to the middle ear and had an outside-in osseous erosion, which was very atypical in otitis media [9]. In case with otitis media, the septa of air cell in the mastoid process and the shape of the middle ear are usually in good condition. It can help us make the differentiation.

In the review of Coutte et al., it was impossible to distinguish CH from LCH on CT findings [18]. Some series reported that LCH lesions can be more destructive [19, 20]. Bilateral involvement does not have any help in distinguishing the two entities. According to our cases, there is something different between LCH and CH. The destruction of LCH is complete, and the outer edge of the mastoid is not continuous. The ossicular chain tends to remain intact in LCH and destructive in CH. In our lesion series, there was one lesion centered in the middle ear which was misdiagnosed as CH, the ossicular chain was intact without any erosion. This sign was not common for CH. Especially the lack of enhancement and restricted diffusion on DWI are the important clues to make the diagnosis as CH.

The extensive bony destruction accompanied with soft mass may suggest a malignant tumor such as rhabdomyosarcoma (RMS). The margin of destruction of RMS tends to be ill-defined, and some residue of small bones can be found in the lesion. From the series of Chevallier et al., which compared the imaging findings of LCH and RMS of the pediatric temporal bone, the subsites of the involvement can be helpful in differentiating LCH from RMS [15]. LCH tend to involve the mastoid process and pars squama of the temporal bone. The anterior portion of temporal which includes petrous apex and middle ear are the most common subsites for RMS.

## Conclusions

The pediatric cases with LCH of the temporal bone have no characteristic clinical features. Bilateral ear involvement can be seen in about half of cases, so the evaluation of both ears is necessary. The ossicular chain usually remains intact and the erosion of otic capsule occurs in about 18.4% of the cases. The imaging finding of pediatric LCH in the temporal bone varies a lot depending on the subsite and extent of the lesion.

### Limitations

Non-uniform imaging techniques are the principal limitation of our study; not every patient obtained thin-slice CT of the temporal bone to visualize the middle and inner ear structures. For the cases who only obtained MRI, the destruction of ossicular chain and otic capsule was just roughly evaluated. Another limitation is the small sample size. A multicenter study is needed to evaluate more cases.

## Abbreviations

CH: Cholesteatoma
LCH: Langerhans cell histiocytosis
RMS: Rhabdomyosarcoma
